# Association of long-term exposure to air pollution with chronic sleep deprivation in adults from 141 urban communities in South Korea: a community-level longitudinal study, 2008‒2016

**DOI:** 10.1017/S2045796021000433

**Published:** 2021-08-05

**Authors:** Whanhee Lee, Daeun Seo, Woojae Myung, Kristi Prifti, Cinoo Kang, Hyemin Jang, Chaerin Park, Michelle L. Bell, Ho Kim

**Affiliations:** 1 School of the Environment, Yale University, New Haven, CT, USA;; 2 Department of Public Health Science, Graduate School of Public Health, Seoul National University, Seoul, Korea; 3 Department of Neuropsychiatry, Seoul National University Bundang Hospital, Seongnam, Korea; 4 Department of Statistics, Ewha Womans’ University, Seoul, Korea; 5 Institute for Sustainable Development, Seoul National University, Seoul, Korea

**Keywords:** Air pollution, chronic sleep deprivation, epidemiological study, sleep duration, urban health

## Abstract

**Aims:**

It has been well known that air pollution and sleep deprivation individually have impacts on human health; however, the association between the two has not been well researched. The aim of this study was to investigate this relationship at a community level.

**Methods:**

We collected sleep outcomes from the Korean Community Health Survey between years of 2008 and 2016. The data contained 1 130 080 selected adults aged ⩾ 19 years, from 141 communities. As sleep outcomes, annual chronic sleep deprivation (% of people who sleep ⩽ 5 h per day on average) and average values of daily mean sleep duration were used. Community-specific annual averages of particulate matter with a diameter ⩽ 10 μm (PM_10_), nitrogen dioxide (NO_2_) and carbon monoxide (CO) were collected and then applied to a linear mixed effects model to estimate the association between air pollution over the past 4 years and sleep outcomes. Population density, green space, health behaviour, and gross regional domestic product per capita variables were considered as confounders in all mixed effect models.

**Results:**

From the linear mixed effect models, we found that the chronic sleep deprivation % was positively associated with PM_10_ (0.33% increase with 95% CI 0.05–0.60; per 10 μg/m^3^) and NO_2_ (0.68% with 95% CI 0.44–0.92; per 10 ppm). Higher PM_10_ and NO_2_ were also associated with shorter sleep duration, with a reduction of 0.37 min (95% CI −0.33 to 1.07 min; per 10 μg/m^3^) and 2.09 min (95% CI 1.50–2.68 min; per 10 ppm), respectively. The associations between PM_10_ and sleep outcomes were higher in females than males and in the older age groups (⩾ 60-years) than in younger age groups (19–39 and 40–59 years). However, the association between NO_2_ and sleep outcomes were more higher in males than in females and in the younger age groups (19–39 years) than other age groups.

**Conclusions:**

Our findings provide epidemiological evidence that long-term interventions to reduce air pollutions are anticipated to provide improvements in sleep deficiency.

## Introduction

Sleep is an integral part of human life and crucial for multiple physiological processes, including modulation of cardiovascular function, restoration of brain energy metabolism and neuronal reorganisation (Wolk *et al*., 2005; Krueger *et al*., 2008; McHill *et al*., 2018). Previous studies reported that sleep deficiency and sleep disorders are associated with an increased risk of overall mortality (Vgontzas *et al*., 2010), as well as cardiovascular diseases (Shahar *et al*., 2001), psychiatric disorders (Gottlieb *et al*., 2003) and autonomic nervous system dysfunctions (Wang *et al*., 2008). In particular, sleep deprivation and other sleep disorders linked to sleep deprivation may cause increased levels of inflammatory markers (Miller and Cappuccio, 2013) and reduced blood flow and oxygen saturation (Netzer *et al*., 1998; Halbower *et al*., 2006), which are commonly related to pulmonary and neurological diseases (Halbower *et al*., 2006; Zanobetti *et al*., 2010). Nevertheless, sleep deficits and disorders, such as sleep deprivation, poor sleep quality, habitual snoring and sleep-disordered breathing, combined with other crucial health problems, are increasingly common (National Sleep Foundation, 2005; Laposky *et al*., 2016).

Air pollution has become one of the world's largest environmental health concerns (Lim *et al*., 2012); about 90% of the world's population lives in regions where air quality exceeds the World Health Organization (WHO) Air Quality Guidelines, and 3 million deaths annually are due to exposure to ambient air pollution (WHO, 2016). Long- or short-term exposure to air pollution may affect the cardiovascular system because of changes on blood markers of systemic inflammation (Pope III and Dockery, 2006; Kahle *et al*., 2015). In addition, previous studies have reported that exposure to air pollution may reduce oxygen saturation and increase inflammatory responses, leading to deterioration of respiratory (Horváth *et al*., 1998; Chiusolo *et al*., 2011) and nervous system (Gerlofs-Nijland *et al*., 2010; Levesque *et al*., 2011).

Sleep and air pollution individually are estimated to have substantial effects on human health; these exposures have typically been studied separately. An association between increased exposure to air pollution and poor sleep quality as well as sleep-related diseases is biologically plausible, however, few studies have investigated the direct effect of air pollution on sleep and sleep disorders (Zanobetti *et al*., 2010; Abou-Khadra, 2013; Fang *et al*., 2015; Lawrence *et al*., 2018). Furthermore, most of these studies were conducted in a single city or community (Fang *et al*., 2015), or in a small number of communities (Zanobetti *et al*., 2010; Lawrence *et al*., 2018). To our knowledge, no studies have provided macro-level evidence for the effects of air pollution on sleep, covering dozens of regions with hundreds of thousands of participants over multiple years.

Thus, this study aimed to investigate whether long-term exposure to air pollution is related to chronic sleep deprivation and sleep duration, using community-level longitudinal data, over 9 years, from 141 communities in South Korea.

## Methods

### Study communities and population

Our study was based on the Korean Community Health Survey (KCHS), which is an annual nationwide community-level health survey started in 2008, by the Korean Center for Disease Control and Prevention (KCDC). The KCHS is a population-based cross-sectional self-reported health survey conducted in adults (aged ⩾ 19 years) living in each of the total 253 communities in South Korea. Communities in the data indicate the local authority districts (comparable to counties of the United States) of residence, as sub-regions of the metropolitan cities and provinces (comparable to states of the United States). The KCHS was designed to estimate annual community-level health-related indicators (e.g. prevalence of disease and health behaviours, such as smoking and drinking %). The KCHS applied two-stage complex, stratified and probability-cluster sampling procedures. In the first stage, smaller sub-districts (tong/ban/li) within each community were randomly selected by the probability proportionate sampling method. Specifically, each community was stratified by dong/eup/myeon, and the tong/ban/li within each dong/eup/myeon was selected as the primary sampling unit. In the next stage, the households within each selected tong/ban/li were also randomly selected using a systematic sampling method, based on the number of adults per household in dong/eup/myeon. If the household was selected, all adults (people aged 19 or more) living in the household were surveyed. The KCHS participants were randomly selected for every year (i.e. the KCHS data do not provide individual follow-up data), and the sample size was approximately 900 participants per community and year (Kim *et al*., 2012). More details are described in the online Supplementary Materials (1. Sampling procedures of the KCHS).

Among the total 253 KCHS communities, we selected the 141 communities with consistent air pollution monitoring during the study period as our study areas. A total of 1 130 080 participants (120 077; 127 509; 126 233; 126 464; 126 293; 126 297; 126 130; 125 715; and 125 362 participants for each year from 2008 to 2016) were included for the whole study period (2008–2016).

### Sleep outcomes

In the KCHS, daily mean sleep duration was recorded as a continues variable in hours, in response to an open-ended question, ‘How many hours do you usually sleep a day?’. In this study, daily mean sleep duration was dichotomised to indicate chronic sleep deprivation as daily mean sleep duration ⩽5 h (Hoevenaar-Blom *et al*., 2011; Fang *et al*., 2015), and crude ratio (%) of chronic sleep deprivation was calculated for each community and each study year. In addition, we used sleep duration as a continuous variable and calculated a simple average value of sleep duration for each community and for each year. In order to investigate differences in population structures among communities, we calculated the age‒sex standardised per cent of chronic sleep deprivation and the individual average sleep duration, based on the 2010 Korean population census (URL: http://kosis.kr). These age‒sex standardised variables were used as additional sleep outcomes in the study. Finally, we calculated age‒sex standardised sleep outcomes by age groups (19‒39 years, 40‒59 years and ⩾ 60 years) and age-standardised sleep outcomes by sex.

### Air pollution data collection

Community-specific air pollution data for ambient particulate matter with diameter ⩽ 10 μm (PM_10_; μg/m^3^), nitrogen dioxide (NO_2_; ppm) and carbon monoxide (CO; ppm) during 2005‒2016 were collected from various monitoring stations. Air pollution data were originally measured hourly. For analysis, we averaged values across all monitoring stations for each year, in each community to generate overall pollution estimates for each community and year. For each community, air pollution variables were measured either from a single or multiple monitoring stations. For communities with multiple monitors, we averaged monitor-specific values to generate community-level estimates.

### Confounders

This study aimed to investigate the association between long-term air pollution and sleep outcomes using regression modelling frameworks. For all regression models, we considered six potential confounders that could simultaneously affect all responses and explanatory variables. Previous studies have shown that urban environments were strongly associated with sleep disorders (Mutatkar, 1995; Rosen *et al*., 2004; Zanobetti *et al*., 2010; James *et al*., 2015; Chambers *et al*., 2016), as well as air pollution (WHO, 2016). Thus, we first considered the annual population density and Normalised Difference Vegetation Index (NDVI) as confounders. NDVI is an estimate of greenness (i.e. vegetation). For every study period, the annual population density was obtained from the database of community health outcomes and health determinants (Kim *et al*., 2018), and the NDVI was obtained from the Moderate Resolution Imaging Spectroradiometer (MODIS; URL: https://modis.gsfc.nasa.gov/) with a 0.05° × 0.05° spatial resolution. We performed re-projection and re-sizing of NDVI gridded estimates to the scale of each community using the *MODIStsp* package in R statistical software.

Second, variables related to health behaviours were collected: age‒sex-stratified body mass index (BMI), prevalence of high-risk smoking (the percentage of people who smoke more than one pack a day on average) and the percentage of high-risk drinking (the percentage of people who drink more than seven (males) or five (females) glasses of alcohol twice or more within a week) (Kim *et al*., 2018). Annual health behaviour-related variables were obtained from the KCHS for each year of the study period and were calculated for overall participants and then stratified by sex and age. Additionally, the variables were standardised by age and sex, except for sex-specific variables (age-standardisation only).

Finally, to consider the average economic status for each community, we used the gross regional domestic product (GRDP) per capita from 2013 to 2015 from the Korea Statistical Information Service (URL: http://kosis.kr). Since the GRDP per capita indicator was not provided annually during the study period, we averaged the annual value of GRDP of 2013‒2015 for each community, using the average value as a confounding variable across the study period. In order to address the right-skewed distribution of the GRDP per capita, this variable was log-transformed before analysis.

### Statistical analysis

We applied linear mixed-effects models (LMEMs) to examine the association between long-term exposure to air pollution and sleep outcomes. We used the 4-year moving averages for each air pollutant (PM_10_, NO_2_, CO) as major exposures. This moving average period was selected based on a previous study (Lawrence *et al*., 2018). For each air pollution type and each sleep outcome, we fitted the LMEM with community-specific random intercepts and with community-specific year trend adjustments, using a natural cubic spline with three equal knots to avoid temporal confounding.

All six confounders were included as linear terms in all LMEMs, and the residual spatial correlation was adjusted by using the longitude and latitude of each community in the model. In addition, the overall temporal trend was also considered, using a natural cubic spline with three equal knots. We estimated the association between each air pollution and each sleep outcome as the change in sleep outcomes per unit change in each air pollution type. All the analytic procedures were repeated for the two-pollutants model and for each subgroup (sex and age).

### Sub-area analysis

Previous studies reported that poor urban living conditions, such as neighbourhood noise, crowded household and less vegetation negatively influence sleep (Mutatkar, 1995; Rosen *et al*., 2004; Zanobetti *et al*., 2010; James *et al*., 2015), thus we hypothesised that the sleep outcomes of people living in more-urban areas would be influenced to a greater extent by air pollution than the sleep outcomes of those living in the less-urban areas. Therefore, we divided our study communities into two sub-categories of urbanicity and conducted a stratified analysis: metropolitan areas (70 communities in the seven metropolitan cities: Seoul, Busan, Daegu, Incheon, Gwangju, Daejeon and Ulsan) and non-metropolitan areas (71 communities not in the seven metropolitan cities). To assess the difference in estimates between metropolitan and non-metropolitan areas, we applied the Wald test (to the null hypothesis that: there was no difference in estimates between the metropolitan and non-metropolitan areas), under the independence assumption (estimates of metropolitan and non-metropolitan areas were mutually independent).

### Sensitivity analysis

The sensitivity of our statistical modelling assumptions was tested by changing the number of previous years considered for air pollution exposure. Other modelling specifications to adjust for the time-trend were also applied.

## Results

**Fig. 1.**
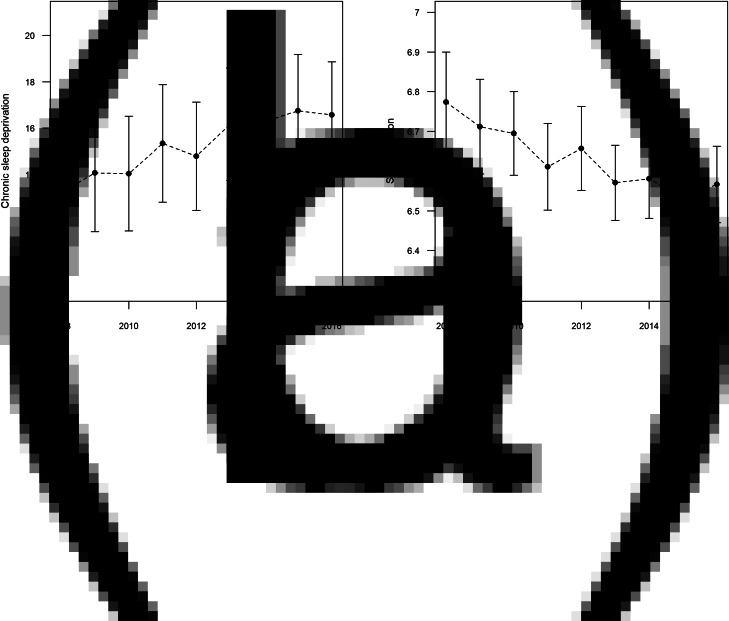
Temporal trend of sleep outcomes; values: mean ± standard deviation. (a) Chronic sleep deprivation: per cent of the population with daily mean sleep duration ⩽5 h. (b) Sleep duration: daily mean sleep duration (hours). Vertical lines represent mean ± standard deviations.

Online Supplementary Figure S1 displays the geographical distribution of the 141 study communities. Figure 1 shows the temporal trend of chronic sleep deprivation and sleep duration in the total participants. The average per cent of people with chronic sleep deprivation decreased over the study period: from 13.2 ± 3.0 (mean ± standard deviation) % in 2008 to 16.6 ± 3.0% in 2016. The average sleep duration also showed a decreasing trend over the study period: from 6.8 ± 0.13 h in 2008 to 6.6 ± 0.10 h in 2016. Online Supplementary Figure S2 presents the age group- and sex-specific time trends of these two sleep outcomes; the trends were generally consistent with those in the overall population.

Table 1 presents descriptive statistics for air pollutants and confounders. Overall, the concentrations of the three types of air pollutants decreased over the study period. In terms of confounders, BMI and prevalence of high-risk smoking decreased over time, whereas the percentage of those with high-risk drinking increased during the study period. Population density and NDVI showed nearly constant temporal patterns throughout the study period. The age group- and sex-specific descriptive statistics for sleep outcomes and health behaviour-related confounders are presented in online Supplementary Table S1. Correlations among air pollutants are displayed in online Supplementary Table S2, with the highest correlation between NO_2_ and CO (0.42).

**Table 1.** Descriptive statistics of air pollutants and confounding variables; mean (standard deviation)

	2008–2010	2011–2013	2014–2016	Total
Air pollution				
PM_10_ (μg/m^3^)	53.0 (7.5)	47.6 (7.9)	47.9 (6.3)	49.5 (7.7)
NO_2_ (ppm)	25.6 (8.3)	24.6 (7.6)	24.3 (7.5)	24.9 (7.8)
CO (ppm)	552.3 (131.4)	529.3 (116.0)	514.5 (105.6)	532.1 (119.1)
Confounder				
Population density (1000 persons/km^2^)	6.7 (7.1)	6.7 (7.0)	6.6 (6.9)	6.7 (7.0)
BMI	24.0 (2.2)	24.6 (2.2)	23.2 (0.2)	23.9 (1.9)
Prevalence of high-risk smoking (%)	2.9 (1.1)	2.3 (1.0)	1.6 (0.8)	2.3 (1.1)
Percentage of high-risk drinking	14.9 (3.5)	16.1 (3.1)	16.9 (2.7)	16.0 (3.2)
NDVI	0.5 (0.1)	0.5 (0.1)	0.5 (0.1)	0.5 (0.1)
GRDP per capita	3.2 (0.7)	3.2 (0.7)	3.2 (0.7)	3.2 (0.7)

*Note*: Values for air pollution averaged across all communities. NDVI is the Normalised Difference Vegetation Index. GRDP per capita is the log-scaled GRDP per capita (Won).

Table 2 shows the long-term associations between air pollution (4-year moving averages) and sleep outcomes in all participants. In the single-pollutant models, higher levels of PM_10_ and NO_2_ were strongly associated with higher percentage of chronic sleep deprivation (increase of 0.33% per 10 μg/m^3^, 95% confidence interval (CI) 0.05–0.60 and 0.68% per 10 ppm, 95% CI 0.44‒0.92, respectively). CO also showed a positive association with chronic sleep deprivation (increase of 0.08%, 95% CI −0.07 to 0.24; per 100 ppm). All air pollutants generally showed negative associations with average sleep duration, more evidently for NO_2_ (change in sleep duration: −2.09 min/day, 95% CI −2.68 to −1.50 min/day; per 10 ppm). Overall, the two-pollutant models yielded similar results; however, the strengths of association between chronic sleep deprivation and PM_10_/CO were decreased after adjusting for NO_2_. Across all models, confounder adjustments reduced the absolute size of associations between air pollution and sleep outcomes; however, directionalities of the associations generally did not change.

**Table 2.** Long-term associations between air pollutants (4-year moving averages) and sleep outcomes

	Change in sleep outcomes (95% confidence internal)	
Pollutant model	Chronic sleep deprivation (%)	Sleep duration (min/day)
PM_10_		
Single	0.33 (0.05 to 0.60)	−0.37 (−1.07 to 0.33)
+ NO_2_	0.24 (−0.02 to 0.50)	−0.12 (−0.78 to 0.54)
+ CO	0.36 (0.08 to 0.63)	−0.43 (−1.14 to 0.27)
NO_2_		
Single	0.68 (0.44 to 0.92)	−2.09 (−2.68 to −1.50)
+ PM_10_	0.65 (0.41 to 0.89)	−2.07 (−2.67 to −1.48)
+ CO	0.69 (0.44 to 0.94)	−2.09 (−2.69 to −1.48)
CO		
Single	0.08 (−0.07 to 0.24)	−0.33 (−0.73 to 0.07)
+ PM_10_	0.10 (−0.05 to 0.26)	−0.35 (−0.75 to 0.04)
+ NO_2_	−0.01 (−0.16 to 0.14)	−0.06 (−0.44 to 0.32)

Chronic sleep deprivation: per cent of the population with daily mean sleep duration ⩽5 h. Sleep duration: daily mean sleep duration. The associations were estimated as the change in sleep outcomes per 10 μg/m^3^ of PM_10_, 10 ppm of NO_2_ and 100 ppm of CO.

**Fig. 2.**
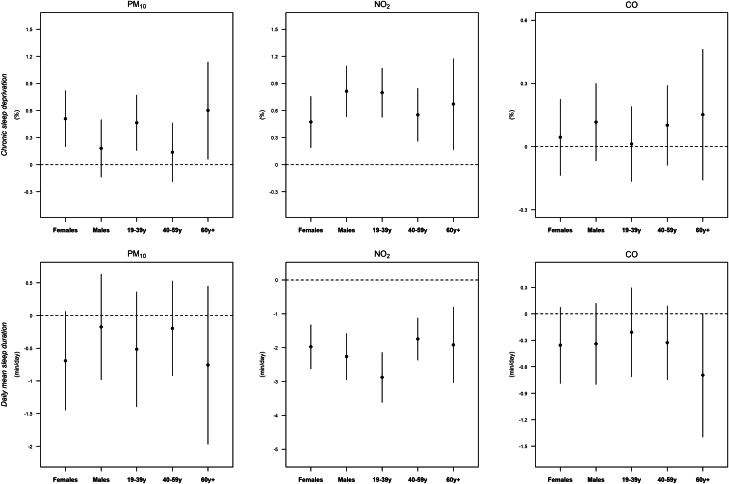
Long-term associations between air pollutants (4-year moving averages) and sleep outcomes for each sub-population. Chronic sleep deprivation: per cent of the population with daily mean sleep duration ⩽5 h. Sleep duration: daily mean sleep duration. The associations were estimated as the change in sleep outcomes per 10 μg/m^3^ of PM_10_, 10 ppm of NO_2_ and 100 ppm of CO.

Figure 2 displays the long-term associations between air pollution and sleep outcomes for each sub-group, based on the single pollutant models. With respect to the central estimates, the positive associations between PM_10_ and sleep outcomes were higher in females than in males, and higher in the ⩾ 60-years and 19‒39-years groups than in the 40‒59-years groups. For NO_2_, based on central estimates positive associations between NO_2_ and sleep outcomes were higher in males and in the 19‒39-years than in females and other age groups, respectively. For NO_2_, associations with both sleep outcomes indicated a trend by age, with higher effects for younger age groups. For CO differences among sub-groups were generally less clear than other pollutants.

Online Supplementary Table S3 displays the descriptive statistics of sleep outcomes, air pollutants and confounders by area type, comparing metropolitan and non-metropolitan areas. Participants living in the metropolitan areas had poorer sleep outcomes than those living in non-metropolitan areas: the average values of chronic sleep deprivation were 15.7% and 14.8% in metropolitan and non-metropolitan areas, respectively. Table 3 shows the long-term associations between air pollution and sleep outcomes by area type, based on the single pollutant models. Overall, the associations between air pollution and sleep outcomes were higher in the metropolitan areas than in non-metropolitan areas, with the most pronounced differences for PM_10_; however, the estimates for metropolitan and non-metropolitan areas were not statistically different.

**Table 3.** Long-term associations between air pollutants (4-year moving averages) and sleep outcomes by metropolitan and non-metropolitan areas

		Change in sleep outcomes (95% confidence internal)					
		Chronic sleep deprivation (%)			Sleep duration (min/day)		
Pollutant	Population	Metropolitan	Non-metropolitan	*p*	Metropolitan	Non-metropolitan	*p*
PM_10_	Total adults	0.66 (0.22 to 1.09)	0.12 (−0.28 to 0.51)	0.072	−0.69 (−1.73 to 0.35)	0.10 (−0.93 to 1.14)	0.29
	Females	0.93 (0.42 to 1.43)	0.33 (−0.12 to 0.78)	0.084	−0.95 (−2.16 to 0.26)	−0.24 (−1.32 to 0.85)	0.387
	Males	0.47 (−0.04 to 0.98)	−0.07 (−0.53 to 0.39)	0.121	−0.52 (−1.70 to 0.65)	0.33 (−0.87 to 1.52)	0.32
	Aged 19–39 years	0.73 (0.22 to 1.23)	0.31 (−0.12 to 0.74)	0.216	−1.12 (−2.43 to 0.20)	0.14 (−1.12 to 1.40)	0.178
	Aged 40–59 years	0.50 (−0.04 to 1.04)	−0.12 (−0.58 to 0.33)	0.083	−0.46 (−1.54 to 0.62)	0.27 (−0.79 to 1.33)	0.341
	Aged 60+ years	1.00 (0.16 to 1.83)	0.35 (−0.46 to 1.15)	0.269	−0.74 (−2.65 to 1.16)	−0.50 (−2.32 to 1.31)	0.859
NO_2_	Total adults	0.98 (0.41 to 1.55)	0.55 (0.27 to 0.84)	0.189	−3.07 (−4.38 to −1.76)	−1.71 (−2.44 to −0.99)	0.075
	Females	0.98 (0.29 to 1.66)	0.32 (−0.02 to 0.66)	0.093	−3.29 (−4.81 to −1.76)	−1.59 (−2.38 to −0.80)	0.053
	Males	0.92 (0.29 to 1.56)	0.71 (0.38 to 1.04)	0.564	−3.00 (−4.46 to −1.55)	−1.88 (−2.71 to −1.05)	0.189
	Aged 19–39 years	0.91 (0.27 to 1.56)	0.65 (0.33 to 0.97)	0.473	−3.66 (−5.27 to −2.05)	−2.57 (−3.46 to −1.67)	0.243
	Aged 40–59 years	1.00 (0.32 to 1.69)	0.36 (0.01 to 0.71)	0.103	−3.34 (−4.60 to −2.08)	−1.05 (−1.81 to −0.29)	0.002
	Aged 60+ years	0.77 (−0.34 to 1.88)	0.70 (0.08 to 1.33)	0.917	−2.01 (−4.48 to 0.47)	−2.03 (−3.43 to −0.64)	0.985
CO	Total adults	0.12 (−0.11 to 0.35)	0.02 (−0.20 to 0.24)	0.54	−0.56 (−1.09 to −0.02)	−0.20 (−0.78 to 0.39)	0.374
	Females	0.04 (−0.24 to 0.31)	0.01 (−0.25 to 0.26)	0.876	−0.59 (−1.21 to 0.03)	−0.24 (−0.85 to 0.37)	0.436
	Males	0.18 (−0.10 to 0.45)	0.01 (−0.24 to 0.27)	0.392	−0.60 (−1.22 to 0.01)	−0.14 (−0.82 to 0.54)	0.319
	Aged 19–39 years	0.02 (−0.25 to 0.29)	−0.04 (−0.28 to 0.20)	0.743	−0.40 (−1.09 to 0.29)	−0.12 (−0.84 to 0.59)	0.58
	Aged 40–59 years	0.12 (−0.17 to 0.41)	0.05 (−0.22 to 0.31)	0.723	−0.59 (−1.16 to −0.02)	−0.21 (−0.82 to 0.39)	0.373
	Aged 60+ years	0.28 (−0.17 to 0.73)	0.04 (−0.40 to 0.49)	0.465	−1.10 (−2.11 to −0.10)	−0.50 (−1.51 to 0.52)	0.404

Chronic sleep deprivation: per cent of the population with daily mean sleep duration ⩽5 h. Sleep duration: daily mean sleep duration. The associations were estimated as the change in sleep outcomes per 10 μg/m^3^ of PM_10_, 10 ppm of NO_2_ and 100 ppm of CO; *p*: *p*-values from the Wald type test (H_0_: estimates between metropolitan and non-metropolitan areas are the same).

Finally, our overall results were robust to sensitivity analyses (online Supplementary Table S4). With changes in the moving average lag years and modelling specifications, results generally remained constantly, suggesting that long-term exposure to air pollution has negative impacts on sleep outcomes.

## Discussion

In this study, the long-term effects of air pollution on chronic sleep deprivation and on sleep duration at a community-level, were investigated using air pollutants (PM_10_, NO_2_ and CO) and health survey data covering 141 communities in South Korea. We found that higher air pollution levels were associated with greater sleep deprivation as well as with shorter sleep duration in the total study population, and that NO_2_ and PM_10_ showed more evident associations with these sleep outcomes than CO.

Several studies have investigated the effects of exposure of a single or of multiple types of air pollutants on overall sleep disorders, sleep-breathing disorder and sleep latency, and have revealed findings consistent with those of our study (Zanobetti *et al*., 2010; Abou-Khadra, 2013; Fang *et al*., 2015; Lawrence *et al*., 2018; Billings *et al*., 2019). A multicentre cohort study in the United States examined whether PM_10_ was related to sleep-disordered breathing in 6441 adults and showed that a higher PM_10_ level was associated with higher respiratory disturbance and with a higher percentage of sleep time at less than 90% molecular oxygen (O_2_) saturation (Zanobetti *et al*., 2010). A study based on the Boston Area Community Health Survey in the United States investigated the association between black carbon (a marker of traffic-related air pollution) and sleep quality in 3821 residents aged 30‒79 years and found that an increase in exposure to black carbon was linked to sleep duration and sleep deprivation (sleep duration ⩽5 h) (Fang *et al*., 2015). A longitudinal study in six cities in the United States examined whether annual mean exposure levels to NO_2_ and PM_2.5_ were related to sleep apnoea in adults aged 45‒84 years, and reported that annual increases in NO_2_ and PM_2.5_ were positively associated with sleep apnoea (Billings *et al*., 2019).

In this study, associations between exposure to air pollution and sleep deprivation/sleep duration suggest trends by age group. Although results are not statistically different, the long-term effects of NO_2_ on sleep outcomes showed a general trend with age, with higher results in younger adults. We considered that this may be associated with indoor/outdoor activity patterns in younger people: in other words, younger adults may be more likely to engage in outdoor and economic activities than other older adults, and thus the average time and amount of exposure to air pollution may also be higher. As the trend by age was strongest for NO_2_ and PM_10_ differential activity patterns by age in relation to traffic may be particularly important. Future research is merited to further explore how air pollution affects sleep in relation to age, and identify the factors and the mechanisms through which the higher air pollution impacts in younger adults.

Our results also provide suggestive evidence that the air pollution-sleep relationship may vary by sex, and that this effect modification varies by air pollutant, however results were generally not statistically different by sex. The central effect estimates of PM_10_ on sleep outcomes were higher in females than in males; however, the effects of NO_2_ were more pronounced in males than in females. Females are generally more sensitive to air pollution than males (Zanobetti *et al*., 2000). We postulate that the indication of different NO_2_ effects by sex may relate to car usage: in South Korea, car usage by males is generally greater than by females (total registered motor vehicles in Seoul, 2016: 1 980 617 for males *v*. 646 634 for females; from the Seoul Open Data Plaza, URL: https://data.seoul.go.kr/).

The estimated effects of air pollution on sleep outcomes were higher in the metropolitan areas than in the non-metropolitan areas, although results were not statistically different. People living in the more-urban areas may generally be more exposed to higher levels of noise, more lights at night and longer working hours than those living in less-urban areas. Our results (online Supplementary Table S3) and the KCDC showed that average sleep duration was generally lower in metropolitan/urban communities than in non-metropolitan/rural communities (Kim *et al*., 2018). In addition, we found that a higher population density and lower greenness were linked to greater chronic sleep deprivation. Our results suggest that the people in the more-urban areas were more susceptible to air pollution. We also found that the average PM_10_ and CO concentrations were lower in metropolitan areas than in non-metropolitan areas. This might be due to thermoelectric power plants and industrial areas, which generate high concentrations of PM_10_ and CO, being located outside metropolitan cities. The lower concentrations in metropolitan areas could be related to higher effects of PM_10_ and CO on sleep outcomes’ previous researchers have reported that the effects of PM on mortality became smaller at high concentrations of PM, and suggested that the ‘saturation effect’ may explain the smaller effects of changes in PM concentration in regions with higher baseline PM levels. Although this study provided limited epidemiological evidence, further investigations should be performed to determine the shape of concentration−response curve for air pollution and sleep outcomes.

This study also showed a long-term lag pattern of air pollution on sleep deprivation and sleep duration. We found that the effects of air pollutants were higher in longer moving average years (3–4 years) than in shorter moving average years (⩽2 years). Prior studies reported that the effects of air pollution on the brain and nervous system may accumulate over a few years and have conjectured that slow rates of translocation of air pollution to the brain or nervous system and its elimination can be a reason (Heusinkveld *et al*., 2016; Weichenthal *et al*., 2020). Thus, most of the air pollution‒sleep studies assumed and investigated the long-term effects of air pollution on sleep; however, the assumed period for the delayed effect varied from 6 months to 5 years (Zanobetti *et al*., 2010; Fang *et al*., 2015; Lawrence *et al*., 2018; Billings *et al*., 2019). These results regarding the ling-term lag-effect of air pollution can be useful for establishing effective sleep and air pollution reduction policies at a community level.

Several experimental and medical studies have reported that sleep deprivation may be related to several chronic cardiovascular, metabolic and neurological disorders with some plausible underlying biological mechanisms: prior studies found that inflammatory markers such as high-sensitivity C-reactive protein and fibrinogen levels increased during sleep deprivation (Meier-Ewert *et al*., 2004; Matthews *et al*., 2010) and with exposure to higher air pollution levels (Pekkanen *et al*., 2000; Ostro *et al*., 2014). Other studies also reported that greater sleep deprivation and air pollution levels might cause disturbance in insulin functions, which is related to an increased risk of metabolic disease (Sun *et al*., 2009; Broussard *et al*., 2012). In addition, previous studies showed that chronic short sleep duration and exposure to air pollution increased neurological disorders (Block and Calderón-Garcidueñas, 2009; McHill *et al*., 2018). Air pollution and sleep-disordered breathing, which may be relevant to sleep deprivation and lower sleep quality, increased the risk of cardiovascular dysfunctions due to hypoxemia and acidosis (Peppard *et al*., 2000; Mehra *et al*., 2006; Zanobetti *et al*., 2010).

The study had several limitations. Firstly, the sleep variables were limited to self-report, since the study was based on a nationwide community health survey on the general health level of the public. In particular, the main disadvantage of this study is the inability to use standardised indicators for measuring sleep deprivation or disorders, such as Pittsburgh sleep quality index, insomnia severity index, actigraphy or polysomnography, which can provide more accurate sleep measurement. Furthermore, measurement of sleep deprivation by sleep duration has limitations, and wakefulness during sleep time or duration of REM sleep should be considered comprehensively. Second, the study results have a limited interpretation with respect to of the individual-level association between air pollution and sleep deprivation. The KCHS was designed to calculate representative values for each community and to identify the spatial and temporal changes in the values (Kim *et al*., 2012); thus, the survey was strictly restricted to using the address of participants and consequently use of individual-level air pollution exposure data is not possible. Therefore, our study results should reflect aggregated community-level results. Third, although some previous studies have reported on the good quality of self-reported surveys and quality control assessments have been conducted for KCHS interviewers and survey results (Lee *et al*., 2016), there may be underlying problems regarding misclassifications and recall bias. Given these limitations, the study needs to be complemented by future clinical or cohort studies with various standardised indicators.

Nevertheless, our study has some key strengths that can offset these limitations. Firstly, this study covered a large representative sample of adults living in urban communities in South Korea, with more than one million participants over 9 years. This large study investigated the effects of air pollution on chronic sleep deprivation and sleep duration. Although there are plausible biological mechanisms, very few previous studies have investigated the association between long-term air pollution and sleep, due to the cost and institutional limitations of precisely measuring sleep and air pollution for many participants. Thus, although our study results have some limitations in terms of clinical interpretations, the study presents a sufficiently valuable assessment of the association between air pollution and general sleep quality at a community-level. We also investigated several regional indicators that might confound assessment of the effects of air pollution on sleep deprivation and sleep duration, and thus our results can be useful in the establishment of public health policies.

In summary, this study examined whether long-term exposure to air pollution was related to sleep deprivation and sleep duration in adults, at a community-level, and found that higher levels of PM_10_, NO_2_ and CO was associated with greater chronic sleep deprivation and with shorter sleep duration. Among air pollution types, NO_2_ showed the highest association with sleep outcomes. In addition, the effects of air pollutions were generally higher in metropolitan areas than in non-metropolitan areas. Our results may provide epidemiological evidence to inform air pollution action plans, and has public health implications for advancing sleep quality.

## Supplementary material

For supplementary material accompanying this paper visit http://dx.doi.org/10.1017/S2045796021000433. click here to view supplementary material
