# Supplementary material for: Association of long-term exposure to air pollution with chronic sleep deprivation in adults from 141 urban communities in South Korea: a community-level longitudinal study, 2008‒2016
Source: Epidemiol Psychiatr Sci. 2021 Aug 5;30:e57. doi: 10.1017/S2045796021000433 (PMC8356258; doi:10.1017/S2045796021000433)
Supplement: Supplementary file 1 [file S2045796021000433sup001.docx]

**Supplementary Materials**

**Title.** Association of Long-term Exposure to Air Pollution with Chronic Sleep Deprivation in Adults from 141 Urban Communities in South Korea: A Community-level Longitudinal Study, 2008‒2016.

**Short title.** Air Pollution and Chronic Sleep Deprivation.

**Authors.** Whanhee Lee, Daeun Seo, Woojae Myung, Kristi Prifti, Cinoo Kang, Hyemin Jang, Chaerin Park, Michelle L. Bell, and Ho Kim

Whanhee Lee and Daeun Seo contributed equally to this research as co-first authors.

**Table of Contents**

1. **Sample design of the KCHS**
2. **Supplementary tables (Table S1-S4)**
3. **Supplementary figures (Figure S1-S2)**
4. **Sampling procedures of the KCHS**
   1. **Sample design**

The sample distribution of the KCHS was based on the two-stage stratification of the surveyed population. The first stratum was “dong/eup/myeon”, which is the sub-administrative area of each community. The second stratum consisted of housing units (apartments and houses). This sample design can mitigate the potential biases that can be caused by random sampling in a small-scale sample survey.^1^

- 1. **Household selection**

The “tong/ban/li” (sub-administrative areas of “dong/eup/myeon”) was selected as the primary sampling unit of housing types in “dong/eup/myeon” through probability proportionate sampling. In this stage, the probability proportionate sampling was based on the registered population size of each “tong/ban/li”.

In the second stage, the number of sampled households in each “tong/ban/li” was counted first, and then the survey households were sampled by systematic sampling based on the number of adults per household in “dong/eup/myeon” where the sampling point is located. Finally, on average, five households were selected as samples in each sampling point, “tong/ban/li”. If the household was selected, all adults (people aged 19 or more) living in the household were surveyed.^1^ On average, approximately 460-500 households were sampled in each of the 253 communities.

- 1. **Response rate**

The KCHS adopted the CAPI (Computer Assisted Personal Interview) system, and the interviewer can start the survey only when the participants agree with their participation. In addition, all interviewers are trained to carry out the survey to the end, the interview takes approximately 20-30 min per person.^1^ If the participants refused to participate in the survey, alternative sampled households were selected. Overall, the rate of substitution was 8.21% (the KCHS 2020 Regular Assessment Report published from the Korea Statistics Promotion Institute).

1. **Supplementary tables**

**Table S1.** Descriptive statistics of sleep outcomes and confounding variables for sub-populations; mean (standard deviation). Chronic sleep deprivation: percent of population with daily mean sleep duration ≤5h. Sleep duration: daily mean sleep duration (hours).

| **Variable** | **Population** | **2008-2010** | **2011-2013** | **2014-2016** | **Total** |
| --- | --- | --- | --- | --- | --- |
| **Sleep deprivation (%)** | Females | 14.8 (3.0) | 16.7 (2.8) | 17.8 (2.7) | 16.4 (3.1) |
|  | Males | 12.7 (3.0) | 14.1 (2.9) | 15.2 (2.8) | 14.0 (3.1) |
|  | Aged 19-39 years | 9.5 (3.0) | 11.1 (3.1) | 12.5 (2.9) | 11.0 (3.2) |
|  | Aged 40-59 years | 13.7 (3.1) | 15.2 (2.8) | 16.3 (2.9) | 15.1 (3.1) |
|  | Aged ≥ 60 years | 22.5 (5.0) | 24.4 (4.6) | 25.1 (4.2) | 24.0 (4.7) |
| **Sleep duration (hour/day)** | Females | 6.7 (0.1) | 6.6 (0.1) | 6.6 (0.1) | 6.6 (0.1) |
|  | Males | 6.7 (0.1) | 6.6 (0.1) | 6.6 (0.1) | 6.6 (0.1) |
|  | Aged 19-39 years | 6.9 (0.1) | 6.8 (0.1) | 6.7 (0.1) | 6.8 (0.2) |
|  | Aged 40-59 years | 6.6 (0.1) | 6.5 (0.1) | 6.5 (0.1) | 6.5 (0.1) |
|  | Aged ≥ 60 years | 6.6 (0.2) | 6.5 (0.2) | 6.4 (0.2) | 6.5 (0.2) |
| **Body Mass Index** | Females | 23.8 (3.4) | 24.8 (3.6) | 22.4 (0.3) | 23.7 (3.0) |
|  | Males | 24.1 (1.4) | 24.3 (1.1) | 24.0 (0.2) | 24.2 (1.1) |
|  | Aged 19-39 years | 23.9 (3.4) | 24.8 (3.3) | 22.8 (0.4) | 23.8 (2.8) |
|  | Aged 40-59 years | 24.2 (1.9) | 24.7 (2.1) | 23.5 (0.2) | 24.1 (1.7) |
|  | Aged ≥ 60 years | 23.8 (2.6) | 23.9 (2.3) | 23.5 (0.3) | 23.7 (2.0) |
| **High-risk smoking (%)** | Females | 0.1 (0.1) | 0.1 (0.1) | 0.1 (0.1) | 0.1 (0.1) |
|  | Males | 5.9 (2.3) | 4.6 (1.9) | 3.3 (1.6) | 4.6 (2.2) |
|  | Aged 19-39 years | 2.1 (1.2) | 1.5 (1.1) | 1.0 (0.8) | 1.5 (1.1) |
|  | Aged 40-59 years | 4.7 (1.9) | 3.7 (1.6) | 2.7 (1.4) | 3.7 (1.9) |
|  | Aged ≥ 60 years | 1.3 (1.0) | 1.1 (0.9) | 0.8 (0.7) | 1.0 (0.9) |
| **High-risk drinking (%)** | Females | 5.2 (2.3) | 5.6 (1.9) | 6.7 (2.1) | 5.9 (2.2) |
|  | Males | 25.0 (5.5) | 27.0 (5.1) | 27.6 (4.2) | 26.5 (5.1) |
|  | Aged 19-39 years | 15.3 (4.2) | 16.3 (3.8) | 17.2 (3.5) | 16.3 (3.9) |
|  | Aged 40-59 years | 17.9 (4.3) | 19.5 (3.9) | 20.5 (3.6) | 19.3 (4.1) |
|  | Aged ≥ 60 years | 8.3 (3.7) | 9.1 (3.3) | 9.5 (2.9) | 8.9 (3.4) |

**Table S2.** Correlations among air pollutants; Pearson’s correlation (*p*-value).

|  | **PM_10_ (µg/m^3^)** | **NO_2_ (ppm)** | **CO (ppm)** |
| --- | --- | --- | --- |
| **PM_10_ (µg/m^3^)** | 1 | 0.26 (0) | 0.17 (0) |
| **NO_2_ (ppm)** | 0.26 (0) | 1 | 0.42 (0) |
| **CO (ppm)** | 0.17 (0) | 0.42 (0) | 1 |

**Table S3.** Descriptive statistics of sleep outcomes, air pollutants, and confounding variables by area: metropolitan areas (70 communities in seven metropolitan cities) and non-metropolitan areas (71 communities not in metropolitan cities); mean (standard deviation). Chronic sleep deprivation: percent of population with daily mean sleep duration ≤5h. Sleep duration: daily mean sleep duration (hours).

|  |  | **Metropolitan**  **areas** | **Non-metropolitan**  **areas** |
| --- | --- | --- | --- |
| **Sleep outcomes** | Chronic sleep deprivation (%) | 15.7 (2.7) | 14.8 (2.8) |
|  | Sleep duration (hour/day) | 6.6 (0.1) | 6.7 (0.1) |
| **Air pollution** | PM_10_ (µg/m^3)^ | 47.6 (6.4) | 51.4 (8.3) |
|  | NO_2_ (ppm) | 26.3 (7.0) | 23.4 (8.2) |
|  | CO (ppm) | 510.3 (106.6) | 553.2 (126.6) |
| **Confounder** | Population density (1,000 persons/km^2^) | 10.6 (7.3) | 2.9 (4.0) |
|  | Body Mass Index | 23.7 (1.6) | 24.1 (2.1) |
|  | Prevalence of high-risk smoking (%) | 2.1 (1.0) | 2.4 (1.2) |
|  | Percentage of high-risk drinking | 15.7 (2.8) | 16.3 (3.5) |
|  | NDVI | 0.4 (0.1) | 0.5 (0.1) |
|  | GRDP per capita | 3.1 (0.8) | 3.2 (0.5) |

Note: Values for air pollution averaged across all communities. NDVI is the Normalized Difference Vegetation Index. GRDP per capita is the log-scaled gross regional domestic product per capita (Won).

**Table S4.** Sensitivity analysis results: the associations between air pollutions (4-year moving averages) and sleep outcomes in adults overall, based on single-pollutant models. Associations were estimated as the increase in sleep outcomes per 10 µg/m^3^ of PM_10_, 10 ppm of NO_2_, and 100 ppm of CO.

|  | **Outcome** | **PM_10_** | **NO_2_** | **CO** |
| --- | --- | --- | --- | --- |
| Main model | Chronic sleep deprivation | 0.33 (0.05 to 0.60) | 0.68 (0.44 to 0.92) | 0.08 (-0.07 to 0.24) |
|  | Sleep duration | -0.37 (-1.07 to 0.33) | -2.09 (-2.68 to -1.50) | -0.33 (-0.73 to 0.07) |
| Current lag (lag 0) | Chronic sleep deprivation | 0.26 (0.01 to 0.51) | 0.34 (0.14 to 0.54) | 0.04 (-0.10 to 0.19) |
|  | Sleep duration | -0.41 (-1.04 to 0.22) | -1.15 (-1.64 to -0.66) | -0.21 (-0.57 to 0.16) |
| 2-year moving average (lag0-1) | Chronic sleep deprivation | 0.40 (0.13 to 0.67) | 0.54 (0.32 to 0.76) | 0.09 (-0.07 to 0.24) |
|  | Sleep duration | -0.64 (-1.32 to 0.03) | -1.71 (-2.24 to -1.17) | -0.39 (-0.77 to 0.00) |
| 4-year moving average (lag0-3) | Chronic sleep deprivation | 0.40 (0.13 to 0.68) | 0.80 (0.54 to 1.06) | 0.15 (-0.01 to 0.30) |
|  | Sleep duration | -0.69 (-1.41 to 0.02) | -2.37 (-3.02 to -1.72) | -0.40 (-0.80 to 0.01) |
| Time trend adjustment:  linear | Chronic sleep deprivation | 0.20 (-0.06 to 0.45) | 0.68 (0.44 to 0.91) | 0.05 (-0.10 to 0.20) |
|  | Sleep duration | 0.40 (-0.28 to 1.08) | -2.10 (-2.69 to -1.51) | -0.07 (-0.47 to 0.33) |
| Time trend adjustment:  df=2 | Chronic sleep deprivation | 0.29 (0.02 to 0.56) | 0.67 (0.44 to 0.91) | 0.07 (-0.08 to 0.23) |
|  | Sleep duration | -0.33 (-1.04 to 0.37) | -2.10 (-2.70 to -1.50) | -0.30 (-0.70 to 0.09) |
| Time trend adjustment:  df=4 | Chronic sleep deprivation | 0.33 (0.06 to 0.60) | 0.66 (0.43 to 0.89) | 0.09 (-0.07 to 0.25) |
|  | Sleep duration | -0.35 (-1.04 to 0.35) | -2.05 (-2.63 to -1.47) | -0.32 (-0.72 to 0.07) |

**Supplementary figures**

**Figure S1.** Geographical distribution of the 141 study communities in South Korea.


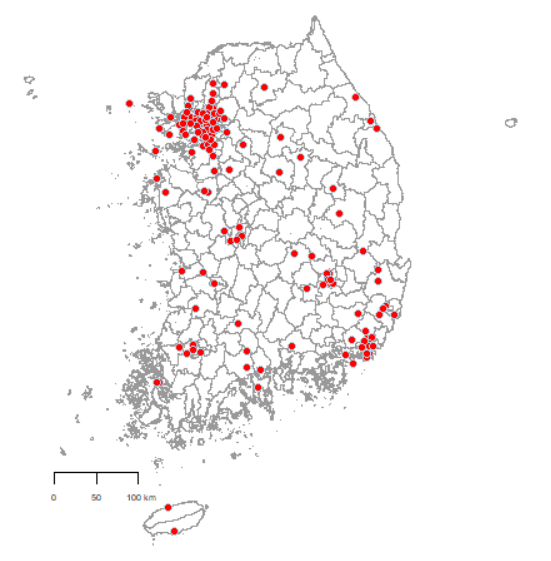


**Figure S2.** Age group- and sex-specific time trends of these two sleep outcomes.


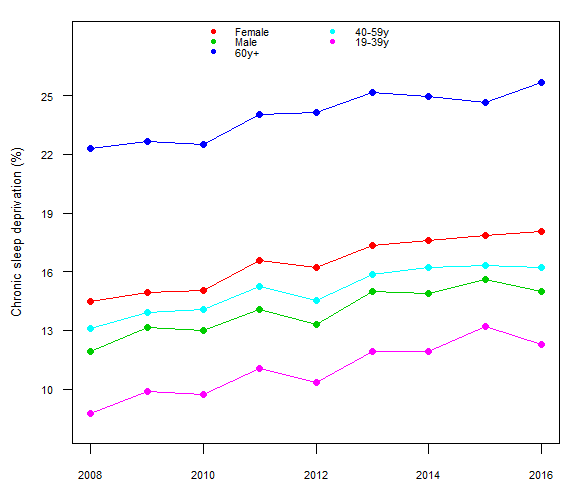


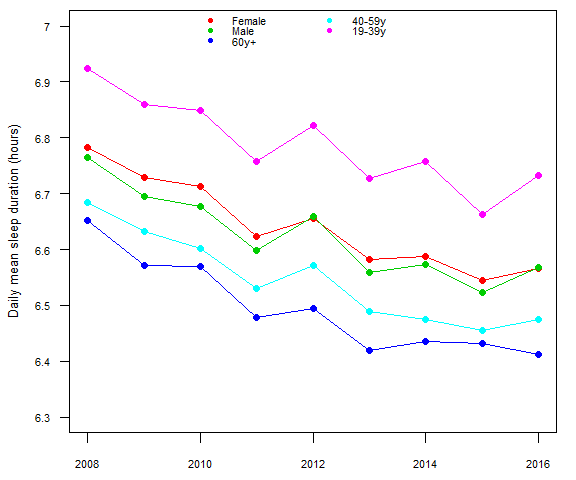


**References for the Supplementary Materials.**

**1.** Kang YW, Ko YS, Kim YJ, et al. Korea community health survey data profiles. Osong public health research perspectives 2015; 6: 211-7.
